# Glomerular C4d in Post-Transplant IgA Nephropathy is associated with decreased allograft survival

**DOI:** 10.1007/s40620-020-00914-x

**Published:** 2020-12-11

**Authors:** Michael Eder, Nicolas Kozakowski, Haris Omic, Christof Aigner, Johannes Kläger, Brian Perschl, Roman Reindl-Schwaighofer, Gregor Bond, Georg A. Böhmig, Željko Kikić

**Affiliations:** 1 Division of Nephrology and Dialysis, Department of Internal Medicine III, Medical University of Vienna, Vienna, Austria; 2 Department of Pathology, Medical University of Vienna, Vienna, Austria; 3 French National Institute of Health and Medical Research, INSERM UMR S1155 “Common and Rare Kidney diseases: from Molecular Events to Precision Medicine”, Tenon Hospital, Paris, France

**Keywords:** Glomerulonephritis, IgA Nephropathy, Graft loss, Kidney allograft, IgAN, C4d, Glomerulus

## Abstract

**Background:**

Glomerulonephritis (GN), including post-transplant IgAN (post-Tx IgAN) is an important contributor to decreased long-term allograft survival. The immunopathological detection of the complement degradation product C4d in glomeruli (C4dG) has been recently described as a risk factor in native kidney IgAN, however little is known about C4dG deposition in post-Tx IgAN. We hypothesized that glomerular C4d may indicate a more aggressive disease course and worse allograft survival in patients with post-Tx IgAN.

**Methods:**

In this retrospective study we assessed the presence and clinical relevance of C4dG in patients with post-transplant IgAN. We analyzed 885 renal allograft recipients, including 84 patients with post-transplant GN. All patients were transplanted between January 1999 and April 2006 and underwent at least one biopsy for differnt causes. The primary endpoint was death-censored graft survival, with a median follow-up of 9.6 (IQR 3.8–13.2) years.

**Results:**

The prevalence of post-Tx GN was 9.5%. Twenty-seven patients with post-Tx IgAN were included. C4dG positive patients (*N *= 18, 66.7%) had significantly worse allograft survival compared to C4dG negative post-Tx IgAN patients and patients without post-Tx IgAN [C4dG positive: 27.8% vs. 55.6% and 66.0%; log-rank: *p *= 0.01]. C4dG remained a significant risk factor (HR 2.22, 95% CI 1.27–3.87) for allograft loss even after adjustment for T cell mediated rejection (TCMR) and antibody mediated rejection.

**Conclusion:**

Glomerular C4d deposition is an independent risk factor for worse graft-survival in patients with post-Tx IgAN, even after adjusting for other risk factors such as antibody mediated rejection.  Assessment of glomerular C4d deposition may provide a valuable prognostic risk assessment tool to identify high risk patients in post-Tx IgAN.

**Graphic abstract:**

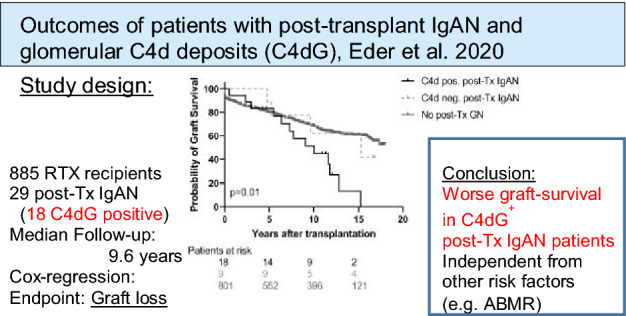

**Electronic supplementary material:**

The online version of this article (10.1007/s40620-020-00914-x) contains supplementary material, which is available to authorized users.

## Introduction

Chronic glomerulonephritis (GN) is one of the leading causes for the development of end-stage renal disease (ESRD) [1]. IgA nephropathy (IgAN) represents the most common primary glomerular disease and its clinical course varies from asymptomatic microhematuria to progressive chronic kidney disease (CKD) and ESRD [2–5]. Aberrant glycosylation of IgA1, circulating antibodies against the galactose-deficient IgA1 and the formation of IgA1-IgG immune complexes, are suggested mechanisms in the so called “multi-hit” hypothesis of IgAN [6–9]. In addition, emerging evidence supports an important role of complement pathways in IgAN [10–19]. Several retrospective studies suggested that glomerular C4d staining (C4dG) may have a prognostic value in native glomerular kidney disease [13, 20–22]. In 2012 Espinosa et al. [21] performed a retrospective multicenter analysis of 283 patients with native kidney IgAN. Around two thirds of their included patients had C4d deposits in glomerular capillaries. C4dG was associated with significantly worse 20-year renal survival (28% vs. 85%) and represented an independent risk factor for progression of CKD in patients with native IgAN [21]. Similar findings were observed in two subsequent analyses, independently from baseline estimated glomerular filtration rate (eGFR) [22, 23].

In renal transplantation, immunopathological C4d staining in peritubular capillaries (C4dPTC) is an accepted marker for the diagnosis of antibody-mediated rejection (ABMR) [24]. Linear C4d deposits in PTC are considered to be induced by HLA-antibodies with subsequent activation of the classical pathway and are associated with a higher risk of allograft loss [25–27]. In native and post-transplant IgA Nephropathy (post-Tx IgAN) similar pathomechanisms have been suggested [28–30], but in contrast to native kidney IgAN, the role of C4dG in post-transplant IgAN is yet to be determined. Further, evidence regarding the frequency and clinical significance of C4dG depositions in post-transplant GN is scarce [31]. We hypothesize that similar to reports in native kidney IgAN [32], C4dG deposits in post-Tx IgAN may indicate worse clinical outcome. Aim of our study was therefore to analyze 1) the frequency of C4dG deposition in allograft recipients with recurrent or *de*-*novo* IgAN, 2) the long-term death-censored graft survival in C4dG positive post-Tx IgAN patients and 3) their clinical course compared to C4dG negative patients.

## Methods

### Study design and patients

The present study was designed as a retrospective single-center analysis. The objective was to assess the presence and clinical relevance of C4dG staining in patients with *de*-*novo* or recurrent post-transplant IgA nephropathy. The primary outcome was death-censored graft-survival, defined as date of initiation of any renal replacement therapy. We analyzed all patients with consecutive renal transplantation between January 1st 1999 and April 1st 2006 (*N *= 1248) and available biopsy results as well as clinical follow-up until January 1st 2017. All biopsies were for cause biopsies, performed upon unexplainable graft dysfunction and/or proteinuria. Study approval was obtained from the local institutional ethics committee (EK-number 1490/2017). All procedures were conducted according to the Declarations of Helsinki and Istanbul.

### Baseline characteristics

Eight hundred and eighty-five patients underwent allograft biopsy for various causes and were included into the study. Baseline characteristics were analyzed from digital patient records and included relevant demographic and transplant-associated findings as well as the occurrence of early (within the first six months after transplant) rejections including T cell mediated rejection (TCMR), ABMR and Borderline lesions and eGFR and proteinuria after one year. The glomerular filtration rate was estimated using the Mayo Clinic formula [33].

### Analysis of biopsies and C4d staining

**Fig. 1:**
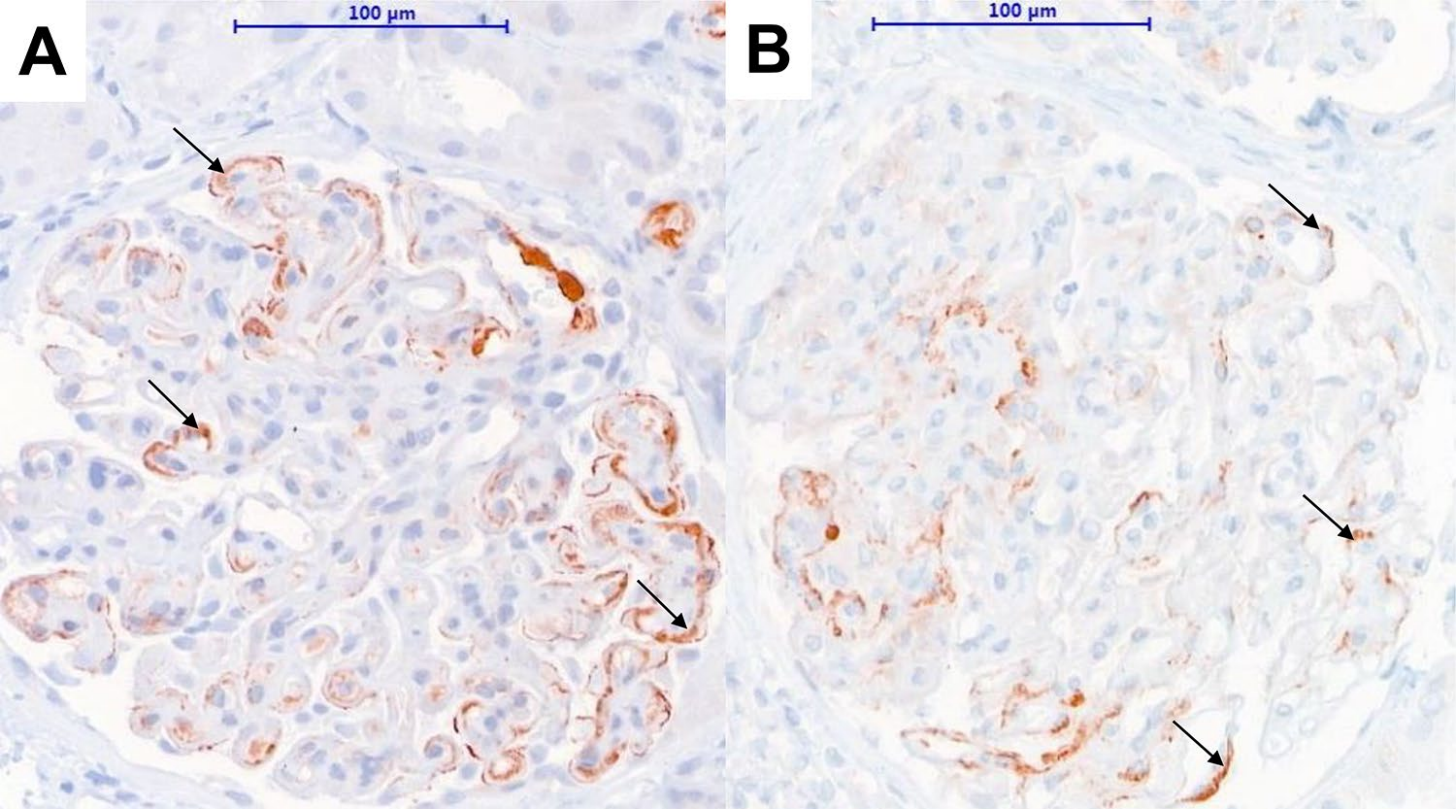
Immunohistochemical staining for C4d deposits. Representative examples of C4d positive post-Tx IgAN patients. Both biopsies show C4d deposits in glomerular peripheral capillary walls. In the first case (**a**), the patient was diagnosed with recurrence of IgAN 8 years after transplantation and lost his allograft within the following 2 years. In (B), the primary underlying renal disease was a not further characterized glomerulonephritis. The patient was diagnosed with post-Tx IgAN 7 years after transplantation and lost his allograft 15 months later. Black arrows indicate granular glomerular C4d in peripheral capillary loops. Magnification scale 400X

Of the 885 included patients, a diagnosis of post-transplant glomerulonephritis was made in 84 (9.5%) of them. Individuals with suspected post-Tx GN underwent detailed immunohistochemistry and ultrastructural analysis with electron microscopy (if material was available). Details on staining procedures and electron microscopy are provided as a supplemental section. The diagnosis of post-Tx IgAN was defined by the presence of glomerular IgA-dominant or co-dominant immune deposits assessed by immunohistochemistry or immunofluorescence, as described before [34]. In the literature the definition of C4dG in native kidney IgAN is variable, depending on the staining technique (IF versus IHC) and includes the mesangium and glomerular capillary walls [14, 16, 17, 19]. Therefore, C4dG deposition was defined as an IHC-based detection of granular C4d deposits in > 5% of peripheral glomerular capillaries or C4d deposits in the mesangium (Fig. 1). All included biopsies with post-Tx GN were retrospectively re-analyzed by two nephropathologists (N.K., J.K.) blinded to clinical data. Biopsies were reassessed according to the 2017 Banff scheme [35]. C4d positive ABMR was considered present if linear, circumferential C4d deposits in PTC were above 0% [36]. Medullary vasa recta were also assessed if available. The diagnosis of C4d negative ABMR had not been established during the observation period for most patients. To accommodate the potential effect of C4d negative ABMR on graft survival, and due to the lack of post-transplant HLA antibody results (which were not standard of care at our center during the study period), we chose a broader definition of the histomorphology compatible for C4d negative ABMR (= suspicion of C4d negative ABMR), which consisted of the presence of inflammatory lesions compatible with ABMR in at least two different renal compartments including a g score > 0, a ptc score > 0, post-transplant thrombotic microangiopathy (TMA), V3 lesions and a cg score > 0. Additionally, following the BANFF guidelines, a ptc score > 0 was not considered sufficient for ABMR diagnosis in the presence of tubulointerstitial infiltrates. Second, g and cg lesions were not scored in the presence of glomerulonephritis. The Oxford criteria (MEST-C score) were applied according to the 2017 published consensus reports [37].

### Treatment

The treatment policy for post-Tx IgAN during the respective time period was watchful waiting and the introduction of angiotensin converting enzyme inhibitor (ACEi)/angiotensin receptor blocker (ARB) (if not already established) in the majority of cases. Only in cases with rapid deterioration of graft function intensified immunosuppressive regimens were applied. Those regimens were: steroid bolus therapy in three patients, switch to calcineurin inhibitors and/or steroid bolus therapy in four patients and one patient received oral cyclophosphamide (2 mg per kilogram). All rejection episodes were treated as follows: patients with TCMR Banff ≥ IA–IIA or Banff Borderline lesions with concomitant allograft dysfunction received a steroid pulse. Steroid-refractory or Banff IIB rejections were treated with anti-thymocyte globulin (ATG) over a course of ten to fourteen days. C4d positive ABMR with allograft dysfunction was treated in the majority of cases as previously described [38].

### Statistical analysis

Continuous variables were expressed as mean and standard deviation or median and interquartile range, whichever appropriate. Categorical variables were expressed as absolute and relative frequencies. Unpaired Student´s t or Mann–Whitney-U was used for comparison of continuous data; group comparisons of categorical variables were analyzed by Fisher´s exact or Chi Squared (*χ*^*2*^) test. Kaplan–Meier analysis and Mantel-Cox log-rank test were used for comparison of graft survival. Graft survival was compared between 1) patients with and without post-Tx GN and 2) between C4dG positive and negative post-Tx IgAN patients and patients without post-Tx GN (control group). For Cox regression analysis, variables differing between the groups with GN and without GN or significantly related to the primary endpoint were included. Variables with *p *< 0.05 in the univariate model were included into the multivariate regression analysis. *p*-values < 0.05 were considered statistically significant. Statistical analysis and graph design were performed using commercially available software systems (Microsoft Office Excel; Microsoft Corp., Redmond, WA; SPSS; Version 25, SPSS Inc., Chicago, IL, GraphPad Prism version 8.40, GraphPad Software, La Jolla, CA, USA).

## Results

### Study cohort

In total 885 patients with indication biopsy and a median follow-up of 9.6/3.8-13.2 years (median/IQR) were included. Most patients were male (63.5%), mean donor and recipient age were 48.7 ± 15.0 and 50.9 ± 13.7 years, respectively. One hundred and two (11.5%) patients received transplants from living donors. Most patients (*N *= 656/74.3%) were on Cyclosporin A-based baseline immunosuppression. Further baseline parameters are shown in Table 1.

**Table 1:** Baseline findings of included patients

Parameters	All patients (*N *= 885)	Glomerulonephritis in biopsy		*P* value
		No GN (*N *= 801)	GN (*N *= 84)	
Female gender, *N* (%)	323 (36.5)	293 (36.6)	30 (35.7)	0.88
Recipient age, mean (SD)	50.9 (13.7)	51.4 (13.6)	46.9 (13.9)	0.008
Donor age, mean (SD)	48.7 (15.0)	48.9 (15.0)	46.8 (15.0)	0.16
Living donor, *N* (%)	102 (11.5)	88 (11.0)	14 (16.7)	0.12
HLA mismatch (A, B, DR), median (IQR)	3 (2–4)	3 (2–4)	3 (2–3)	0.28
Years of follow-Up, median (IQR)	9.6 (3.8–13.2)	9.9 (3.6–13.3)	8.3 (4.7–11.5)	0.52
eGFR 1 year after Tx, ml/min/1.73 m^2^, median (IQR)	53 (33–75)	52 (34–70)	53 (33–75)	0.060
Protein/creatinine ratio 1 year after Tx, mg/g, median (IQR)	77 (0–237)	70 (0–235)	104 (0–312)	0.26
Re-transplantation, *N* (%)	162 (18.3)	134 (16.7)	28 (33.3)	< 0.001
Cold ischemia time in hours, median (IQR)	13 (8–19)	13 (8–19)	11 (6–16)	0.010
DGF, *N* (%)	194 (22.3)	178 (22.7)	16 (19.0)	0.45
Immunosuppression				
Initially Tacrolimus, *N* (%)	148 (16.8)	135 (16.9)	13 (15.7)	0.78
Initially Ciclosporin, *N* (%)	656 (74.3)	590 (73.8)	66 (79.5)	0.25
Initially MMF, *N* (%)	827 (94.1)	750 (94.2)	77 (92.8)	0.59
Initially AZA, *N* (%)	14 (1.6)	13 (1.6)	1 (1.2)	0.77
Rejections				
Early C4d pos. ABMR, *N* (%)	105 (11.9)	95 (11.9)	10 (12)	0.96
Early C4d neg. ABMR*, *N* (%)	77 (8.7)	59 (7.4)	18 (21.4)	< 0.001
Early Banff Borderline, *N* (%)	133 (15.0)	125 (15.6)	8 (9.5)	0.14
Early TCMR Banff ≥ 1, *N* (%)	270 (30.5)	246 (30.7)	24 (28.6)	0.69
Underlying renal disease				
Diabetic nephropathy, *N* (%)	103 (11.6)	100 (12.5)	3 (3.6)	0.015
Cystic kidney disease, *N* (%)	101 (11.4)	92 (11.5)	9 (10.7)	0.83
Glomerulonephritis, *N* (%)	200 (22.6)	163 (20.3)	37 (44.0)	< 0.001
IgA-nephropathy, *N* (%)	51 (5.8)	41 (5.1)	10 (11.9)	0.011
Vascular nephropathy, *N* (%)	54 (6.1)	52 (6.5)	2 (2.4)	0.14
Chronic pyelonephritis, *N* (%)	45 (5.1)	40 (5.0)	5 (6.0)	0.70
Obstructive nephropathy, *N* (%)	19 (2.1)	18 (2.2)	1 (1.2)	0.53
Other, *N* (%)	97 (11.0)	87 (10.9)	10 (11.9)	0.77
Unknown, *N* (%)	266 (30.1)	249 (31.1)	17 (20.2)	0.039

*ABMR* antibody-mediated rejection, *AZA* azathioprine, *DGF* delayed graft function, *GN* glomerulonephritis, *IQR* interquartile range; *MMF* mycophenolate mofetil; *N* number, *Neg.* negative, *SD* standard deviation, *TCMR* T cell mediated rejection, *Tx* transplantation, *Pos.* positive. Mayo Clinic estimation was used to calculate the eGFR. Early rejection was classified as occurrence within the first six months after transplantation; *Suspicion of C4d negative ABMR, which consisted of the presence of at least two lesions being compatible with ABMR including a g score > 0, a ptc score > 0, post-transplant thrombotic microangiopathy (TMA), V3 lesions and a cg score > 0

### Patients with post-Tx GN

Biopsy confirmed post-transplant glomerulonephritis was diagnosed in 84 patients within 3/1–6 (median/IQR) years after transplantation, representing a prevalence of 9.5%. Post-Tx IgAN was found in 34 (40.5%) patients. Patients with post-Tx GN were significantly younger (46.9 ± 13.9 vs. 51.4 ± 13.6 years; p = 0.008) at transplantation and had more often received a prior allograft (33.3% vs. 16.7% in patients without GN, *p *< 0.001). Glomerulonephritis was the major cause for end-stage renal disease in the post-Tx GN group (44.0% vs. 20.3% in patients without post-Tx GN, *p *< 0.001, Supplemental Table 1). Recurrence of primary glomerular disease was assumed in eighteen (21.4%) patients. There were no patients with IgA vasculitis (Purpura-Schönlein-Henoch) as native kidney disease.

### Post-transplant Glomerulonephritis is associated with graft survival

**Fig. 2:**
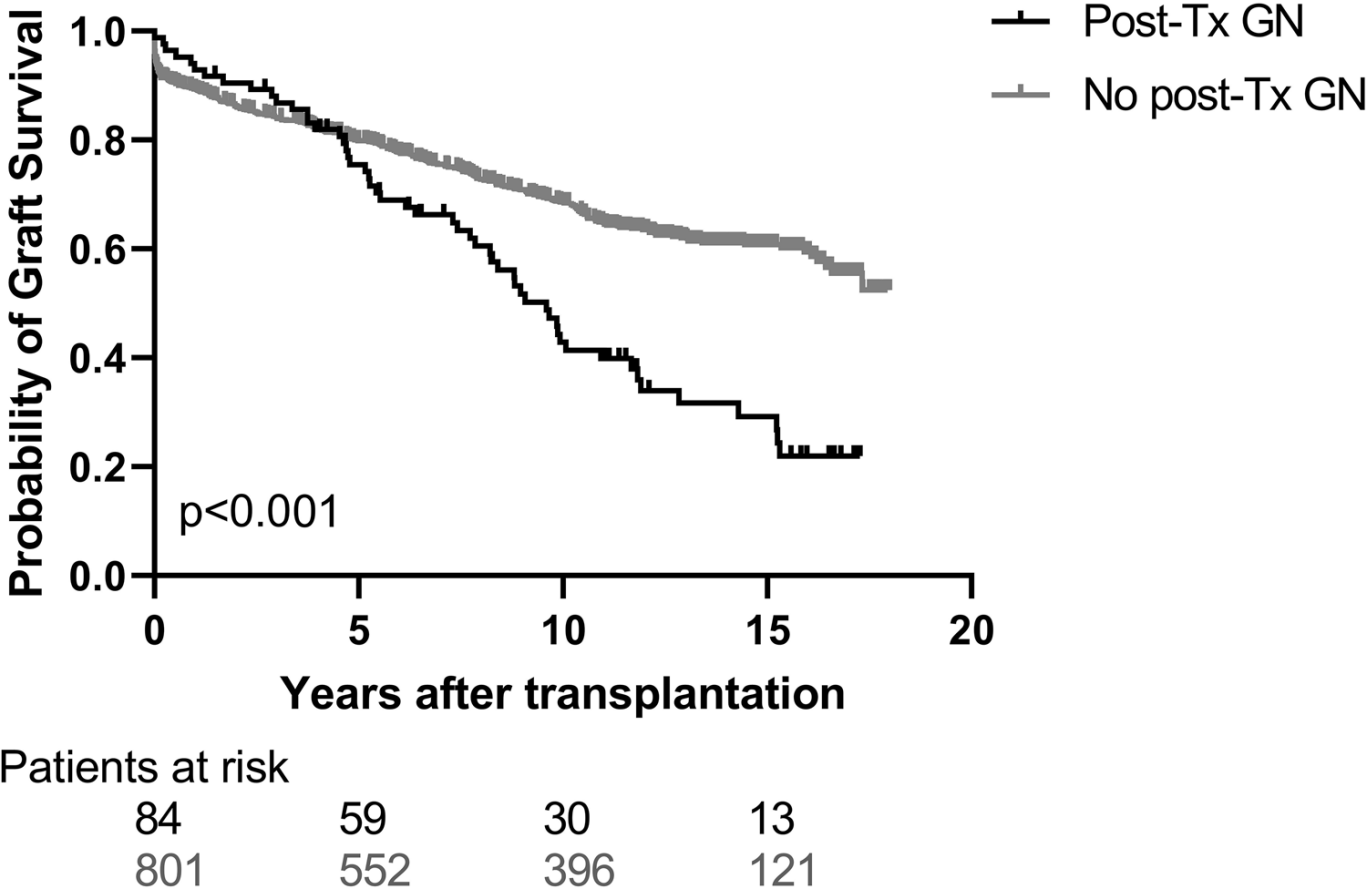
Kaplan-Meier allograft survival in relation to the presence of post-Tx GN. Groups included were patients with glomerulonephritis after transplantation (*N *= 84, black solid line) and patients without glomerulonephritis after renal transplantation (*N *= 801, grey solid line). Log-Rank test revealed significant, worse allograft survival in patients with post-Tx GN (*p* < 0.001)

Figure 2 shows KM graft survival of patients with post-Tx GN compared to all patients without post-Tx GN. Death-censored graft survival was significantly worse in the post-Tx GN group (36.1% vs 66%; log-rank test *p *< 0.001). Univariate Cox-regression revealed a two-fold increase in the risk for graft loss in patients with post-Tx GN [HR 2.1; (95% CI 1.4–3.2); *p *< 0.001].

### Post-transplant IgA nephropathy (post-Tx IgAN)

Thirty-four patients (26.7% female) were diagnosed with post-Tx IgAN within 3.3/1.4–7.1 years (median/IQR) after transplantation. Recurrence of disease was suspected in nine (26.5%) patients and was significantly more frequent in living donor recipients [57.1% (4/7) vs. 18.5% (5/27) in deceased donor recipients, p = 0.039]. The risk for recurrence of IgAN was not associated with pre-sensitization or early C4d negative ABMR. None of the included patients received a steroid-sparing immunosuppressive regimen. Serum creatinine at the time of index biopsy was 2.0/1.7–2.6 mg/dl (median/IQR) and did not significantly differ from other post-Tx GN patients [2.0/1.6–2.7 mg/dl (median/IQR), *p *= 0.26]. Twenty (66.7%) patients were on ACE-inhibitors or ARBs. Further baseline characteristics are shown in Table 2. KM death-censored graft survival was significantly worse in patients with post-Tx IgAN compared to patients without post-Tx GN [38.2% vs. 66.0%, log-rank test *p *= 0.004, univariate Cox-regression: HR 2.2; (95% CI 1.5–4.9); *p *= 0.001]. Death-censored graft survival was not significantly different between *de*-*novo* or recurrent IgAN (log-rank test, *p *= 0.93).

**Table 2:** Characteristics of all 34 post-Tx IgAN patients

Parameters	Patients with post-Tx IgAN (*N *= 34)
Female gender, *N* (%)	9 (26.7)
Donor age, median (IQR)	49 (37–56)
Age at transplantation, median (IQR)	48 (32–57)
HLA-mismatch, median (IQR)	3 (2–3)
Living donor transplantation, *N* (%)	7 (20.6)
First transplantation, *N* (%)	27 (79.4)
Second transplantation, *N* (%)	6 (17.6)
Recurrence of disease, *N* (%)	9 (26.5)
Histological findings in index biopsies	
Interstitial Inflammation (i), median (IQR)	1 (0–1)
Tubulitis (t), median (IQR)	1.5 (0–2)
Intimal arteritis (v), median (IQR)	0 (0–0)
Arteriolar hyalinosis (ah), median (IQR)	1 (0–2.8)
Interstitial fibrosis (ci), median (IQR)	2 (1–2)
Tubular atrophy (ct), median (IQR)	1 (1–2)
Mesangial matrix expansion (mm), median (IQR)	2 (1–2)
Active crescents, *N* (%)	2 (5.9)
Renal parameters at the time of biopsy	
Creatinine at biopsy, mg/dl, median (IQR)	2.0 (1.7–2.6)
Protein/creatinine ratio mg/g, median (IQR)	2.2 (0.6–4.6)
eGFR ml/min/1.73 m^2,^ median (IQR)	37.1 (23.7–48.6)
Creatinine 1 year after biopsy, mg/dl, median (IQR)	2.4 (1.6–4.1)
Years after transplantation, median (IQR)	3.3 (1.4–7.1)
Years of follow-up duration, median (IQR)	9.4 (5.6–11.8)

*eGFR* estimated glomerular filtration rate, *HLA* human leukocyte antigen, *IQR* interquartile range, *N* number, *Post-Tx IgAN* post-transplant IgA nephropathy, Mayo Clinic estimation was used to calculate the eGFR

### Immunohistochemistry in post-Tx IgAN patients

C4dG stainings were available in 27 patients with post-Tx IgAN. Of those, 18 (66.7%) showed positive C4dG staining. In most cases (*N *= 16), granular C4dG was found in peripheral capillary loops. Four patients had mesangial C4d deposits (two of them had mesangial and peripheral capillary C4d deposits). Glomerular basal membrane splitting was found in five out of eleven patients with available silver staining and peripheral capillary C4d deposits. In three biopsies concomitant C4d positive ABMR (linear C4d positive in PTC) was diagnosed. Those patients were diagnosed with chronic antibody-mediated rejection. Simultaneous tubulointerstitial infiltrates were observed in fourteen patients (BANFF ≥ 1 *N *= 7, BANFF Borderline *N *= 7). Neither the Oxford MEST-C single scores nor the sum score differed significantly between C4dG positive- and negative patients (data not shown). Crescents were found in two patients with post-Tx IgAN. As shown in Table 3, neither baseline variables nor laboratory findings at the time of index biopsy, including graft function and proteinuria, differed significantly between C4dG positive and negative post-Tx IgAN patients. Moreover, intensified immunosuppression as a treatment of post-Tx IgAN was rare and did not differ between both groups (data not shown). C4dG positivity was a constant finding in follow-up biopsies (available in six C4dG positive patients). All patients had positive C4dG stainings in follow-up biopsies (three with mesangial and three patients with peripheral capillary C4d deposits).

**Table 3:** Patient characteristics in relation to the presence of glomerular C4d

Parameters	All patients (*N *= 27)	C4dG in patients with post-Tx IgAN		*p* value
		Positive (*N *= 18)	Negative (*N *= 9)	
Donor age, median (IQR)	49 (37–57)	47 (36–57)	51 (46–55)	0.31
Age at transplantation, median (IQR)	51 (39–58)	50 (32–56)	53 (47–58)	0.28
HLA-mismatch, median (IQR)	3 (2–4)	3 (2–4)	3 (2–3)	0.71
Rejections				
Early C4d pos. ABMR, *N* (%)	3 (11.1)	2 (11.1)	1 (11.1)	> 0.99
Early C4d neg. ABMR*, *N* (%)	8 (29.6)	7 (38.9)	1 (11.1)	0.14
Early Banff Borderline, *N* (%)	1 (3.7)	1 (5.6)	0	0.47
Early TCMR Banff ≥ 1; *N* (%)	9 (33.3)	7 (38.9)	2 (22.2)	0.39
Findings at the time of index biopsy				
Years after transplantation, median (IQR)	3 (1.6–6.8)	2.9 (1.6–6.8)	3.7 (2.3–5.3)	0.940
Creatinine at biopsy, mg/dl, median (IQR)	2.0 (1.7–2.6)	2.1 (1.7–2.7)	2.0 (1.9–2.6)	0.93
eGFR 1 year after biopsy, ml/min/1.73 m^2^, median (IQR)	35.6 (23.3–45.5)	33.4 (23.8–50.5)	37.0 (22.6–40.0)	0.59
Protein/creatinine ratio mg/g, median (IQR)	2100 (700–4300)	3300 (400–4600)	2100 (1200–2200)	> 0.99
ACEi/ARB at biopsy, *N* (%)	16/23 (69.6)	11/15 (73.3)	5/8 (62.5)	0.59
Number of antihypertensive drugs, median (IQR)	3 (3–4)	3 (3–4)	3 (3–4)	0.59
More than three antihypertensives, *N* (%)	9/23 (39)	7/15 (46.7)	2/8 (25)	0.31
Oxford MEST-C				
MEST-C sum score, median (IQR)	1 (0.3–2)	1 (0.3–2)	1 (0.8–1.3)	0.78
Immunohistochemistry findings				
IgM mesangial, *N* (%)	27 (100)	18 (100)	9 (100)	N/A
IgM peripheral, *N* (%)	12 (44.4)	8 (44.4)	4 (44.4)	> 0.99
C3 mesangial, *N* (%)	18 (66.7)	11 (61.1)	7 (77.8)	0.39
C3 peripheral, *N* (%)	5 (18.5)	4 (22.2)	1 (11.1)	0.48
C1q mesangial, *N* (%)	26 (96.3)	17 (94.4)	9 (100)	0.47
1q peripheral, *N* (%)	11 (40.7)	8 (44.4)	3 (33.3)	0.58
Outcomes				
Follow-Up time in months, median (IQR)	103.2 (81.1–119.1)	98.9 (75.2–118)	103.6 (82.6–141.6)	0.43
Death during follow-up, *N* (%)	11 (40.7)	8 (44.4)	3 (33.3)	0.58
Underlying renal disease				
Glomerulonephritis, *N* (%)	12 (44.4)	8 (44.4)	4 (44.4)	> 0.99
IgA Nephropathy, *N* (%)	8 (29.6)	4 (22.2)	4 (44.4)	0.23
Diabetic Nephropathy, *N* (%)	1 (3.7)	1 (5.6)	0	0.47
Vascular Nephropathy, *N* (%)	1 (3.7)	1 (5.6)	0	0.47
Cystic kidney disease, *N* (%)	2 (7.4)	2 (11.1)	0	0.30
Unknown, *N* (%)	7 (25.9)	5 (27.8)	2 (22.2)	0.76

*ABMR* antibody-mediated rejection, *ACEi* angiotensin converting enzyme inhibitor, *ARB* Angiotensin receptor blocker, *eGFR* estimated glomerular filtration rate, *HLA* human leukocyte antigen, *IQR* interquartile range, *N* number, *neg.* negative, *pos.* positive, *Post-Tx IgAN* post-transplant IgA nephropathy, *TCMR* T-cell mediated rejection. Early rejection was classified as occurrence within the first 6 months after transplantation; the antihypertensive treatment was known in 23 patients; Mayo Clinic estimation was used to calculate the eGFR; *: Suspicion of C4d negative ABMR, which consisted of the presence of at least two lesions being compatible with ABMR including a g score > 0, a ptc score > 0, post-transplant thrombotic microangiopathy (TMA), V3 lesions and a cg score > 0

### C4dG is associated with worse death-censored graft survival

**Fig. 3:**
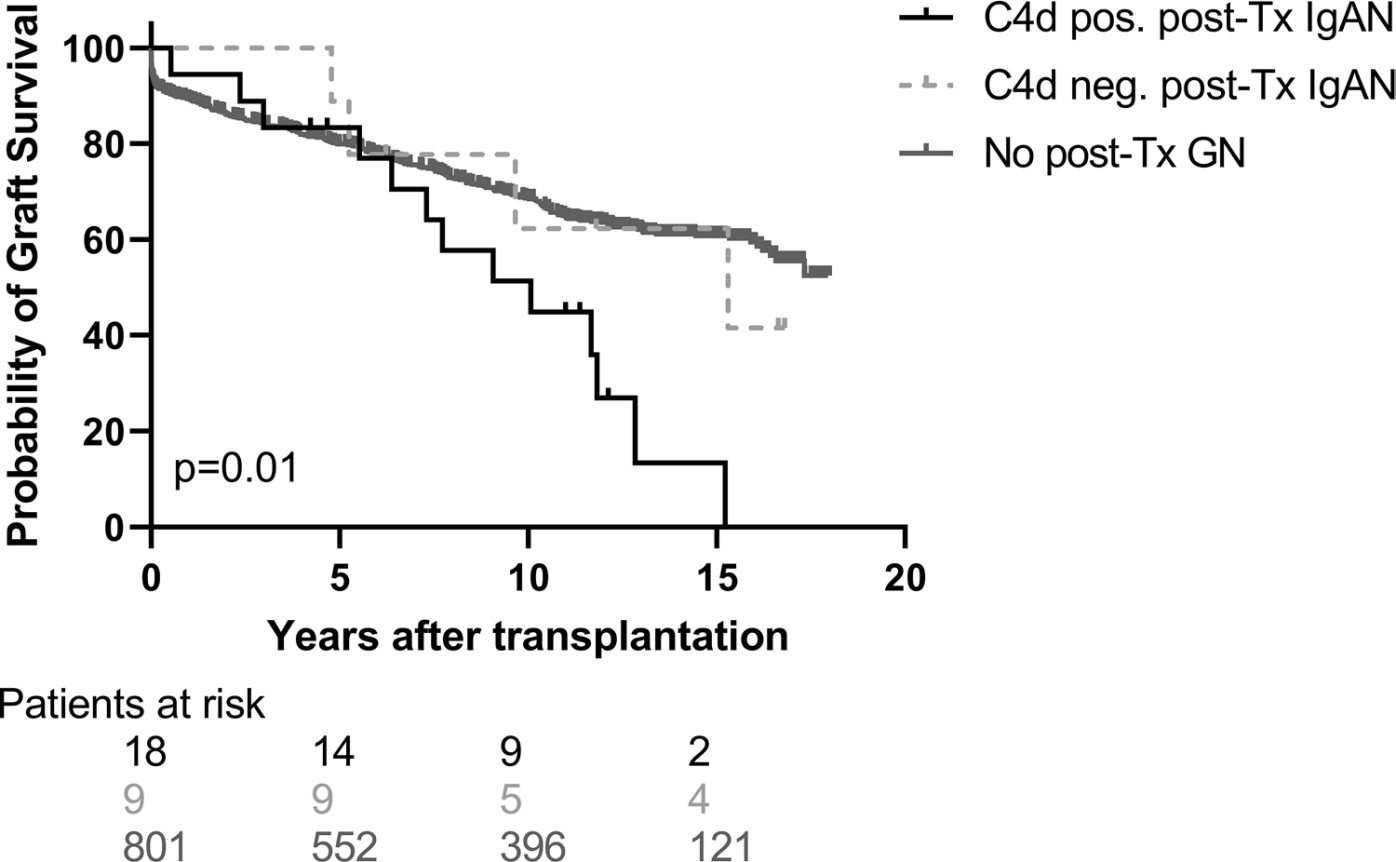
Kaplan–Meier allograft survival curves in relation to glomerular C4d staining and diagnosis of post-Tx IgAN. Groups included were glomerular C4d positive post-Tx IgAN patients (*N *= 18, black solid line), glomerular C4d negative post-Tx IgAN patients (*N *= 9, grey dashed line) and patients without post-Tx GN (*N *= 801, grey solid line). Log-Rank test showed significant allograft-survival between these three groups (*p* = 0.01)

Kaplan Meier analysis revealed a significantly worse death-censored allograft survival of C4dG positive post-Tx IgAN patients [death-censored graft-survival: C4dG 27.8% vs. 55.6% in C4dG negative post-Tx IgAN patients and 66.0% in patients without GN; log-rank: *p *= 0.01] (Fig. 3). Exclusion of the three patients with concomitant C4d positive ABMR did not materially change the highly significant association of C4dG with graft loss (data not shown). In univariate Cox-regression analysis, C4dG positive IgAN represented a highly significant risk factor for allograft loss [HR 2.22 (95% CI 1.27–3.87), *p *= 0.005]. C4dG positive post-Tx IgAN remained independently associated with death-censored graft-loss (HR 3.57, 95% CI 1.80–7.09, *p *= 0.001) even after adjustment for multiple baseline covariates including ABMR, Banff Borderline lesion and TCMR (Table 4).

**Table 4:** Cox regression model in relation to death-censored graft loss: univariate and multivariate analysis in all 883 patients

Parameters	Univariate analysis		Multivariate Analysis	
	HR (95% CI)	P-value	HR (95% CI)	*P* value
Reference: No glomerulonephritis	–	–	–	–
C4dG positive post-Tx IgAN	2.22 (1.27–3.87)	0.005	3.57 (1.80-7.09)	0.001
C4dG negative post-Tx IgAN	1.15 (0.43–3.08)	0.79	–	–
Living Donor	0.74 (0.52–1.06)	0.10	–	–
Re-transplantation	1.66 (1.29–2.13)	< 0.001	1.10 (0.69–1.74)	0.69
HLA-mismatch, per number	1.08 (1.00–1.17)	0.048	1.07 (0.95–1.25)	0.31
Sensitized patients	1.54 (1.21–1.97)	< 0.001	1.46 (0.99–2.15)	0.059
Donor Age in years	1.01 (1.01–1.02)	0.001	1.00 (0.99–1.02)	0.31
Cold ischemia time	1.01 (1.00–1.03)	0.046	1.00 (0.97–1.02)	0.76
eGFR after 1 year, per ml/min/1.73 m^2^	0.95 (0.95–0.96)	< 0.001	0.97 (0.96–0.98)	< 0.001
Protein/creatinine quotient > 0.5 g after 1 year	2.80 (1.92–4.09)	< 0.001	2.52 (1.60–3.95)	< 0.001
Early Banff Borderline	0.62 (0.44–0.88)	0.008	0.83 (0.50–1.37)	0.46
Early TCMR Banff ≥ 1	1.50 (1.20–1.88)	< 0.001	1.17 (0.82–1.66)	0.39
Early C4d positive ABMR	1.78 (1.32–2.38)	< 0.001	1.16 (0.78–1.74)	0.47
Early C4d negative ABMR*	2.03 (1.47–2.80)	< 0.001	2.73 (1.73–4.31)	< 0.001
Underlying renal disease IgAN	0.90 (0.57–1.44)	0.67	–	–
Underlying renal disease diabetic nephropathy	0.80 (0.54–1.18)	0.26	–	–

*ABMR* antibody-mediated rejection, *GN* Glomerulonephritis, *post-Tx IgAN* post-transplant IgA nephropathy; *HLA* human leukocyte antigen, *eGFR* estimated glomerular filtration rate (Mayo Clinic formula). Early rejection was classified as occurrence within the first 6 months after transplantation, *: Suspicion of C4d negative ABMR, which consisted of the presence of at least two lesions being compatible with ABMR including a g score > 0, a ptc score > 0; post-transplant thrombotic microangiopathy (TMA), V3 lesions and a cg score > 0

## Discussion

In this analysis of a representative and well characterized cohort of renal transplant recipients, we demonstrated the clinical relevance of glomerular capillary C4d staining as a profound prognostic marker in patients with post-Tx IgA nephropathy. To the best of our knowledge, this is the first study showing that post-Tx IgAN patients with C4dG deposits had significantly worse death-censored allograft survival. The substantially increased risk for allograft loss remained significant even after adjustment for other known risk factors such as HLA-mismatch, donor age and the occurrence of biopsy-proven rejection. The prognostic impact of C4dG in post-Tx IgAN is in line with descriptions of patients with native kidney IgAN, where C4dG depositions have been associated with an overall worse renal prognosis [15, 21, 22, 39]. However, in renal transplant cohorts with post-transplant glomerulonephritis the interpretation of immunopathological C4dG may be hampered due to HLA-antibody-triggered C4dPTC and/or C4dG. In our cohort only three subjects were diagnosed with concomitant ABMR, we can therefore safely exclude ABMR as a driving force of increased risk for graft loss in post-Tx IgAN patients. Nevertheless, the underlying pathomechanisms are still not fully understood. It was hypothesized that more progressive forms of IgAN are associated with endocapillary hypercellularity and vascular injuries, leading to complement activation and deposits of complement degradation products in various glomerular compartments—in particular in mesangial cells and glomerular capillary walls [40]. Recent studies in native kidney IgAN however have suggested additional clinical relevance of C4d staining in other renal compartments including tubules, interlobular arteries and arterioles [14, 16, 17, 19].

Graft loss occurred in two thirds of our group of C4dG positive post-Tx IgAN patients, within a median of two years after the index biopsy. At the time of index biopsy, neither graft function nor proteinuria differed between C4d positive- and negative IgAN patients. Based on our findings, we therefore hypothesize that C4dG may help to identify a progressive course of disease in patients with post-Tx IgAN, even before a severe deterioration of graft function is observed. This is in contrast to studies in native kidney IgAN [21, 39]. In 2014, Espinosa et al. [21] reported on 283 patients with IgAN (38.5% C4d positive). In their study, patients with C4dG positive biopsies already had significantly more proteinuria and worse eGFR at the time of index biopsy. We believe that the difference in our findings may be due to regular follow-ups of transplant recipients and thus earlier diagnosis of post-Tx IgAN compared to native kidney IgAN patients with very heterogeneous disease courses. Nevertheless, in both studies the association of C4dG with ESRD remained statistically significant even after correction for baseline eGFR. In contrast, in our study C4dG negative patients had death-censored graft survival that was comparable to the control group, suggesting an acceptable allograft prognosis even after post-Tx IgAN diagnosis. Most of our patients were not subjected to intensified immunosuppression after the diagnosis of post-Tx IgAN. Moreover, concomitant TCMR and Banff Borderline lesions were routinely treated with steroid boli, and their prevalence was not different between the two groups (data not shown). We can therefore safely exclude a relevant treatment bias between C4dG positive and negative groups.

Another finding that needs to be emphasized is that post-transplant glomerulonephritis was a major contributor for decreased long-term graft survival. Not only post-Tx IgAN patients but all patients with a diagnosed post-Tx GN had significantly worse death-censored graft-survival compared to the control group. This is in line with previously published findings from Chailimpamontree et al. [41].

One major strength of this study is the long follow-up period. We believe this is essential because post-Tx GNs often manifest late and show a heterogeneous disease course. Consequently, insufficient follow-up periods may lead to an underestimation of the decreased long-term survival of post-Tx GNs. Further, multiple relevant risk factors for decreased allograft survival, including donor-associated risk factors, were available and were included into the multivariate analysis.

Our study has limitations that need to be discussed. First, though the study design was retrospective, we have included a large sample of post-transplant biopsies with long-term follow-up and corrected our findings for multiple covariates including HLA-mismatch, and early- and concomitant rejections. Moreover, the follow-up period of included biopsies was comparable between C4dG positive and negative patients, arguing against a relevant time selection bias. We found predominantly peripheral capillary- rather than mesangial C4d positivity, a deposition pattern that may seem counterintuitive at first glance. Nevertheless, our findings are in concordance with previous reports from patients with native IgAN, where C4d was described in various glomerular compartments: Rath et al. reported that 40% of his patients with IgAN showed C4d depositions in the glomerular capillary walls [17]. These findings were in line with a report from Meng et al. [16] where C4d was found in the mesangium as well as in capillary loops. Differences in IgA and C4d staining patterns may be related to possible alterations of non-endothelial C4d deposits resulting from formalin-fixation or paraffin-embedding [42]. Moreover, despite equally distributed mesangial- and peripheral C1q deposition, a potential role of MBL pathway activation cannot be excluded in certain patients. Another important limitation is the lack of systematic post-transplant assessment of anti HLA- antibodies. We have corrected this by using a broad definition of histological features compatible with C4d negative ABMR, and by the inclusion of both C4d positive and C4d negative ABMR in multivariate models. However, we cannot completely rule out the possibility that some biopsies were not attributed to ABMR.

Further, it is important to know that isolated microhematuria after transplantation was not considered a biopsy indication in the study period.

In conclusion, this study shows a prognostic value of C4dG deposition in patients with post-transplant IgAN. C4dG positivity was significantly associated with graft loss and remained an independent risk factor even after correction for covariates in the multivariate analysis. These findings are of clinical importance providing new evidence and suggesting that C4dG assement may be useful for a risk-based management of post-Tx IgAN patients.

## Electronic supplementary material

Below is the link to the electronic supplementary material.

Electronic supplementary material 1 (DOCX 13 kb)

## Abbreviations

ABMR: Antibody-mediated rejection
ACE: Angiotensin converting enzyme
ACEi: Angiotensin converting enzyme inhibitor
ARB: Angiotensin receptor blocker
ATG: Anti-thymocyte globulin
AZA: Azathioprine
C4d: Complement split product C4d
C4dG: Complement split product C4d in glomeruli
C4dPTC: Complement split product C4d in peritubular capillaries
CI: Confidence interval
ci: Interstitial fibrosis
CKD: Chronic kidney disease
ct: Tubular atrophy
DGF: Delayed graft function
eGFR: Estimated glomerular filtration rate
ESRD: End-stage renal disease
Gd-IgA1: Galactose-deficient IgA1
GN: Glomerulonephritis
HLA: Human leukocyte antigen
HR: Hazard ratio
IAS: Immunoadsorption
IHC: Immunohistochemistry
IgAN: IgA nephropathy
IQR: Interquartile range
KM: Kaplan–Meier
MMF: Mycophenolate mofetil
post-Tx GN: Post-transplant Glomerulonephritis
post-Tx IgAN: Post-transplant IgA nephropathy
PTC: Peritubular capillaries
SD: Standard deviation
TCMR: T cell mediated rejection
TMA: Thrombotic microangiopathy
Tx: Transplantation
