# Supplementary material for: Glomerular C4d in Post-Transplant IgA Nephropathy is associated with decreased allograft survival
Source: J Nephrol. 2020 Dec 11;34(3):839–49. doi: 10.1007/s40620-020-00914-x (PMC8192385; doi:10.1007/s40620-020-00914-x)
Supplement: Supplementary file 1 — Electronic supplementary material 1 (DOCX 13 kb) [file 40620_2020_914_MOESM1_ESM.docx]

**Supplemental material: Immunostaining procedures and electron microscopy**

Immunohistochemistry was performed using polyclonal antibodies against Anti-IgG (DAKO, policlonal rabbit anti human,REF A0423; dilution 1:5000); Anti-IgM (DAKO, polyclonal rabbit anti human, REF A0425; dilution 1:900); Anti-IgA (DAKO, polyclonal rabbit anti human, REF A0262; dilution 1:2500) Anti-C3c (DAKO, polyclonal rabbit anti human, REF A0062; dilution 1:250); Anti-C1q (DAKO, polyclonal rabbit anti human, REF A0136; dilution 1:600) incubation 1 hour at room temperature. Antigen retrieval (AR) was performed with enzymatic digestion 13 minutes at 37°C in water bath (Protease XIV, Fa. SIGMA, P5147-5G). Slides were stained with AEC (BD Pharmingen, Cat: 551015) following the kits instructions, counterstained with Mayer's hemalum and mounted. The hole procedure was done with the UltraVision LP Large Volume Detection System HRP Polymer (Thermo Scientific, REF TL-125-HL) following the kits instructions.

Electron microscopy was performed as followed: Biopsys were fixed in 4% paraformaldehyde (Merck, Germany) in 0,1m cacodylate buffer (pH 7,2, Sigma Aldrich, Germany). Tissue was postfixed with 1% OsO4 (Electron Microscopy Sciences, USA) in 0,1m cacodylate buffer (pH 7,2, Sigma Aldrich, Germany). Tissue was than stained with 1% aqueous uranyl acetate (Serva, Germany). Biopsy specimen were embedded in epoxy resin (Serva, Germany). Ultrathin sections were arranged on an Ultramicrotome (Ultracut-E, Reichert – Jung, Austria, or Ultracut UC6, Leica, Germany). Final staining procedure was done with uranyl acetate (Serva, Germany) in methanol (Fisher Scientific, Germany) and lead citrate (Merck, Germany). The sections were then analyzed with the JEOL JEM-1010 or the JEOL JEM-1400 plus transmission electron microscope (JEOL, Japan).
